# New Iflavirus Species Characterized from Mosquitoes Captured in the Sao Paulo Zoological Facilities

**DOI:** 10.3390/microorganisms12091749

**Published:** 2024-08-23

**Authors:** Lilian de Oliveira Guimarães, Santana Lobato Bahia, Geovani de Oliveira Ribeiro, Endrya do Socorro Foro Ramos, Fabiola Villanova, Vanessa dos Santos Morais, Juliana Telles-de-Deus, Vanessa Christe Helfstein, Jesus Maia dos Santos, Ramendra Pati Pandey, Xutao Deng, Eric Delwart, Vera Lucia Fonseca de Camargo-Neves, Antonio Charlys da Costa, Karin Kirchgatter, Élcio Leal

**Affiliations:** 1 Área Técnica de Doenças Vinculadas a Vetores e Hospedeiros Intermediários, Instituto Pasteur, São Paulo 01027-000, SP, Brazil; lilianguima@gmail.com (L.d.O.G.);; 2 Institute of Biological Sciences, Federal University of Pará, Belem 66077-830, PA, Brazil; 3 General-Coordination of Public Health Laboratories, Health and Environment Surveillance Secretariat, Ministry of Health, Brasilia 70723-040, DF, Brazil; 4 Department of Cellular Biology, University of Brasilia (UNB), Brasilia 70910-900, DF, Brazil; 5 Laboratory of Virology (LIM 52), Instituto de Medicina Tropical, Universidade de São Paulo, São Paulo 05403-000, SP, Brazilcharlysbr@yahoo.com.br (A.C.d.C.); 6 School of Health Sciences and Technology (SoHST), UPES, Bidholi, Dehradun 248007, UK, India; 7 Vitalant Research Institute, San Francisco, CA 94143, USA; 8 Department Laboratory Medicine, University of California San Francisco, San Francisco, CA 94143, USA; 9 Programa de Pós-Graduação em Medicina Tropical, Faculdade de Medicina, Instituto de Medicina Tropical, Universidade de São Paulo, São Paulo 05403-000, SP, Brazil

**Keywords:** metagenomics, virome, *Culex*, *Anopheles*, *Aedes*, insect-specific viruses, mosquitoes

## Abstract

Metagenomic studies of mosquito viromes demonstrated a more diverse composition than just an exclusive composition of pathogenic arboviruses transmitted to humans. In our study, the virome of 866 female mosquitoes collected throughout 2020 at the São Paulo Zoo, located in the city of São Paulo/SP—Brazil, was obtained. Specifically, in this paper, we describe a new virus found by viral RNA extraction and next-generation MiSeq sequencing of a group of 23 specimens of *Anopheles* (*Nys*.) *strodei*. The complete genome with a length of 9709 nucleotides was characterized by a positive orientation and a single strand, with a single large ORF, which encodes a polyprotein of 2987 amino acids. The phylogenetic analysis showed an association with the viral family *Iflaviridae* and the *Riboviria* realm. We carried out comparisons with translated sequences of the capsid regions of other iflavirus, and the identities in relation to our sequence were below the minimum limit of 90%, indicating that possibly it is a new species of iflavirus. Our findings contribute to expanding knowledge of virome composition among mosquito species in Brazil and globally. Moreover, we provide a viral genome reference specific to this geographic region and *Culicidae* family of mosquitoes. This resource facilitates future in silico recognition and assembly of viral genomes within metagenomic datasets.

## 1. Introduction

The viral diseases transmitted by mosquitoes to humans, including dengue, chikungunya, zika, yellow fever, West Nile fever, Japanese encephalitis, etc., are considered of great importance for global public health, due to the high number of cases and the large number of countries affected [1,2,3,4]. This scenario has attracted, in recent decades, a large amount of research focusing on arboviruses, and viruses transmitted by arthropods such as mosquitoes [5,6]. This increase in research related to arboviruses, combined with advances in Next Generation Sequencing (NGS) technologies and bioinformatics analyses, has enabled the discovery of a variety of new viruses that do not necessarily infect humans, but, for example, constitute new insect-specific viruses (ISVs), such as most iflaviruses [7,8,9,10].

The iflaviruses (a word derived from the name “*Infectious flacherie virus*” of the type species of the *Iflaviridae* family) are a small group of viruses, represented by a single genus (*Iflavirus*), with 16 described species, belonging to the *Iflaviridae* Family. In the Taxonomic System of the International Committee on Viral Taxonomy (ICTV), the Family *Iflaviridae* is placed in the realm *Riboviria*, kingdom *Orthornavirae*, phylum *Pisuviricota*, class *Pisoniviricetes*, and order *Picornavirales* [11].

The *Iflaviridae* Family brings together viruses whose viral particles do not have an envelope and are approximately spherical, with icosahedral symmetry with diameters ranging from 22 nm to 30 nm. Genomes are represented by a single-stranded positive RNA molecule (9 to 11 kb), which has only one open reading frame (ORF) encoding a polyprotein approximately 3000 amino acids long. A Genome-Bound Viral Protein (VPg) is covalently attached to the 5′ end of the genomic RNA while the 3′ end is polyadenylated. There are two non-coding regions (5′NCR and 3′NCR) each being positioned at one of the ends flanking the ORF, and the sizes of the NCRs vary between iflavirus species. Proteolytic cleavage of the polyprotein produces all viral proteins. In the N-terminal region of the polyprotein, we find structural proteins of the capsid, a small leader protein followed by the proteins VP2, VP4, VP3 and VP1, respectively. The VP2, VP3 and VP1 proteins form the external portion of the capsid, measuring between 28 kDa and 44 kDa, while the VP4 protein, located more internally, is smaller, measuring between 4 kDa and 12 kDa [11,12]. In the C-terminal region of the polyprotein, non-structural proteins involved in the replication and processing of the polyprotein are located, these are an RNA helicase [13], a cysteine protease similar to 3C [14], and an RNA-dependent RNA Polymerase (RdRP) [15].

Translation of the polyprotein can be initiated at an internal ribosome entry site (IRES) in the 5′ non-coding region (5′NCR), it serves as an initiator for the translation of the polyprotein, which is probably a common mechanism in the genus [16,17]. The three nonstructural proteins exhibit conserved, defining domains.

All reported hosts of iflaviruses are arthropods, mainly insects of various orders, such as mosquitoes, aphids, leafhoppers, flies, bees, ants, silkworms, and wasps. Several iflaviruses are economically important because they are highly pathogenic to honey bee species [18], and hosts such as silkworm [19] and other iflaviruses appear to cause little or no symptoms. However, even for those that only infect mosquitoes (ISVs—insect-specific viruses), and thus have no direct impact on public or veterinary health, understanding them is of paramount importance, as several studies have indicated that these viruses can be used to interfere with the replication of pathogenic arboviruses of humans and animals of economic importance in insect hosts, representing a biological strategy to reduce the occurrence of arboviruses [20,21,22].

In Brazilian territory, there is a wide variety of arboviruses circulating in its main Biomes, among which the Amazon Forest, the Atlantic Forest and the Cerrado stand out. In the Amazon Forest region alone, more than 210 arboviruses have already been identified, among which the Ilhéus [23], Rocio [24], Mucambo [25] and West Nile [26] viruses can be cited. However, intense deforestation in the Amazon rainforest and other Brazilian biomes may be contributing to a greater spread of arboviruses [2]. In this context, there is a need to increase surveillance of emerging viruses in the main Brazilian biomes and their transition areas. Metagenomic studies in mosquitoes can contribute to monitoring the circulation of pathogenic viruses and the discovery of new virus species, enabling a greater understanding of viral evolution, as well as strategies for controlling pathogenic arboviruses and their resulting diseases.

In this study, we employed a metagenomics, bioinformatics, and phylogenetic approach to analyze the virome of adult female mosquitoes from the *Culicidae* family, collected in São Paulo, Brazil. We collected 866 female mosquitoes throughout 2020 from the São Paulo Zoo to explore their virome. Our research resulted in the genetic and phylogenetic characterization of the genome of a newly identified iflavirus, providing insights into its phylogenetic relationships and genomic features.

## 2. Materials and Methods

### 2.1. Mosquito Collection

Mosquitoes were collected at the Coordenadoria de Fauna Silvestre (ex-Fundação Parque Zoológico de São Paulo, FPZSP), located in the city of São Paulo/SP, Brazil. The collections were carried out on 5 July 2020, from 6 to 9 October 2020, 3 November 2020 and 1 December 2020. Except for 5 July (winter), all collections were carried out in spring. A total of 16 traps were installed with luminous bait and carbon dioxide attractant in eight points (Bosque das Aves—23°38′54.0″ S/46°37′13.4″ W; Enclosure 113—23°38′58.3″ S/46°37′03.9″ W; Corridor 61—23°39′01.5″ S/46°37′03.0″ W; Lake Bridge—23°39′08.1″ S/46°37′03.9″ W; Enclosure 69—23°39′11.2″ S/46°36′59.7″ W; Lake 70—23°39′04.0″ S/46°37′11.9″ W; Flamingo Enclosure—23°38′55.2″ S/46°37′16.8″ W; and Ponto Extra—23°38′48.2″ S/46°37′14.4″ W), distributed within the park. The collection points were the same as those used by Guimarães and collaborators (2021) [27]. Each point received two CDC traps (Centers for Disease Control—miniature light traps) installed on two levels (ground and canopy), using light (white or UV) and carbon dioxide attractants. The traps were exposed for 12 h between afternoon and morning twilight. The sampled insects were frozen alive in liquid nitrogen, placed in duly identified cryotubes (Product Number 430659, Corning^®^, Corning, NY, USA), and transported to the laboratory, where they were stored in a −80 °C freezer. Information from each collection was recorded in a specific bulletin including the technique used, climatic variables, location, date, number of tubes and collection period.

### 2.2. Identification of Mosquito Species

Non-engorged adult females were morphologically identified on a cold table at −20 °C with a stereoscope, using taxonomic keys proposed by Forattini (2002) [28], Consoli and Lourenço de Oliveira (1994) [29], and Lane (1953) [30], in Pasteur Institute of São Paulo. Pools of up to 10 mosquitoes were prepared according to species, date, collection point, and location. For metagenomic analysis, 866 female mosquitoes were selected and distributed into 25 groups (Meta 1 to Meta 25), belonging to the genera *Culex*, *Aedes,* and *Anopheles*. In this study, we specifically described the analysis of group number 20 (Meta 20), containing 23 specimens of *Anopheles (Nys.) strodei*.

### 2.3. Sequencing

The group of mosquitoes number 20 (Meta 20) was homogenized for 60 sec at 1500 rpm in a FastPrep-96 homogenizer (MP Biomedicals, Irvine CA, USA) using 900 μL of Hanks’ buffered salt solution (HBSS) and 1 g of ceramic beads, followed by centrifugation for 10 min at 14,000 rpm at 4 °C. Approximately 300 µL of the supernatant was filtered through a 0.45 µm filter (Merck Millipore, Billerica, MA, USA) in order to remove insect debris. The filtered sample was treated with nucleases (7 µL of TURBO DNase and 3 µL RNase Cocktail Enzyme Mix-Thermo Fisher Scientific, Waltham, MA, USA) and followed by viral RNA extraction using the Maxwell 16 Viral Total Nucleic Acid Purification kit (Promega, Inc., Madison, WI, USA) according to the manufacturer’s instructions. For reverse transcription, 10 µL of viral RNA was incubated with 3 µL of random hexamer (20 s at 95 °C, 20 s at 90 °C, 20 s at 85 °C, 20 s at 80 °C and 3 min at 75 °C) and then added 4 µL of Superscript 5× buffer, 2 µL of dNTP (10 mM), 1 µL DTT (0.1 M), 1 µL of Superscript III RT enzyme (200 U) (Thermo Fisher Scientific, Waltham, MA, USA). The solution was incubated for 15 min at 25 °C, 120 min at 50 °C, 15 min at 70 °C, 5 min at 4 °C, 2 min at 95 °C and maintained at 4 °C. The second strand of cDNA was synthesized by adding 1 U of DNA Polymerase I Large (Klenow) Fragment (Promega Inc., Madison, WI, USA), 0.5 mM dNTPmix and 1X Reaction Buffer to sample and incubating for 120 min at 37 °C and 75 °C for 20 min to inactivate the enzyme. DNA fragments between 500 and 1000 nucleotides were selected using the ProNex^®^ Size Selection kit and used to prepare the DNA library using the Nextera XT DNA library preparation kit (Illumina Inc., San Diego, CA, USA) according to the manufacturer’s instructions. Sequencing was carried out on the next-generation MiSeq sequencer (Illumina Inc., San Diego, CA, USA) at the Central Laboratory of the Hospital das Clínicas of the University of São Paulo.

### 2.4. Bioinformatics and Phylogenetic Analysis

Using the Galaxy Europe Server, the raw data obtained from NGS was subjected to quality control analysis using the FASTQC Tool v0.12.1 and then processed to remove adapters and low-quality readings by performing the Trimmomatic program with the following parameters: LEADING:3, TRAILING:3, SLIDINGWINDOW:4:20, MINLEN:36 and TruSeq2-PE as adapter file to be removed, with the data once again subjected to quality control through FASTQC. De novo assembly was performed using the SpadesRNAviral v3.15.5 [31], MetaSpades [32], and MEGAHIT v1.2.9 [33] assemblers, all with parameters in default mode. All contig sequences obtained from the three assembly tools mentioned above were grouped using the CD-HIT-EST tool v4.8.1 with the following parameters: -c 0.98 -G 0 -As 0.9 -g 1 -n 9, to exclude redundant sequences, having ≥ 98% identity. The sequenced viral contigs were identified using DIAMOND v2.1.9 [34] by performing a BLASTx with the recovered viral proteins. Therefore, the contigs were compared using the BLASTx and BLASTp tools for similarity with viral proteins based on nucleotides and polyprotein, respectively, in the GenBank genetic sequence database (http://www.ncbi.nlm.nih.gov, accessed on 11 March 2024). The cognate sequence with the highest identity in BLASTx and BLASTp (with the highest percent of sequence similarity ever deposited in GenBank), together with a sequence from the same species, were selected, as well as 41 cognate sequences with a minimum identity of 50% as well as the reference sequences of the Family Iflaviridae (17 sequences) suggested from the ICTV website (https://ictv.global/report/chapter/iflaviridae/iflaviridae/iflavirus) (accessed on 11 March 2024) and downloaded into the GenBank database. E values (e-value) were defined in each search to reduce the number of random matches.

The translated sequence extracted from Mosq_B20_SP01 was compared with the translated sequences of the closest viral members available at NCBI by multiple sequence alignment (MSA) using the “MUSCLE” algorithm implemented in MEGA software (Version 11) [35]. The phylogenetic trees were constructed using the maximum likelihood method [36], in the IQ-TREE v.1.6.12 program [37] with the statistical support of an ultrafast bootstrap [38] with 1200 replications. FigTree v.1.4.4 (available at http://tree.bio.ed.ac.uk/software/figtree/) (accessed on 11 March 2024) was used to visualize the phylogeny.

### 2.5. Genomic Annotation

The Mosq_B20_SP01 polyprotein ORF was predicted using the online tool ORFfinder v0.4.3 (accessed on 11 March 2024) [39]. The domains and motifs of the polyprotein were predicted using the Conserved Domains Tool v0.4.4 [40,41,42] and Motif Finder (https://www.genome.jp/tools/motif/, accessed on 11 March 2024), respectively.

### 2.6. Probability Mapping

To explore phylogenetic content, we used a likelihood mapping approach to a set of aligned sequences. The likelihood is based on the analysis of the maximum probabilities for the three fully resolved tree topologies that can be computed for four random sequences. The three probabilities are represented as a point within an equilateral triangle. The central region of the triangle represents star-like (unresolved) trees, vertices demonstrate well-resolved phylogenies, and regions connecting the vertices represent the situation in which it is difficult to distinguish between two of the three trees. The positioning of the likelihoods in the triangle indicates the phylogenetic pattern in the alignment. Thus, likelihood mapping can be used to some posteriori test the phylogenetic signal and confidence of an internal branch in a phylogenetic tree. Likelihood mapping was performed using the IQ-TREE v.1.6.12 software.

### 2.7. Genetic Distance

Genetic distance and its standard error were calculated using the maximum likelihood plus gamma correction and bootstrap model with 1200 replications. Distances were calculated using the MEGA software (Version 11) using the pairwise method. The identities of nucleotides and amino acids were obtained using the SDT Program version 1.2 [43]. Estimation of similarity alignments for each unique pair of sequences was performed using algorithms implemented in MUSCLE. After calculating the identity score for each pair of sequences, the program uses the NEIGHBOR component of PHYLIP to calculate a tree. The rooted neighbor-joining phylogenetic tree orders all sequences according to their likely degrees of evolutionary relatedness. The results are presented in a frequency distribution of paired identities in a graphical interface.

## 3. Results and Discussion

### 3.1. Characterization of Mosquito Sampling and Genus

The Meta 20 group was collected from the soil of the following locations: Bosque das Aves, Enclosure 69, Ponte do Lago, Enclosure Flamingos, Enclosure 113, Extra, and Corridor 61. This sample presented only individuals of the genus *Anopheles* identified in the species *Anopheles (Nyssorhynchus) strodei*.

### 3.2. Genome Annotation of Mosq_B20_SP01 Identified in Anopheles

**Figure 1:**
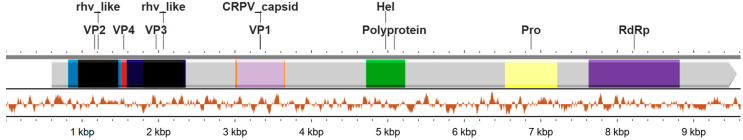
Regions of the translated genome of the complete genome sequence obtained in this work. The Mosq_B20_SP01 genome is represented by a single strand of positive RNA, consisting of a single ORF coding for a polyprotein, which when processed produces structural proteins (VP2 in light blue, VP4 in red, VP3 in dark blue and VP1 colored in orange), as well as non-structural proteins (Helicase colored green, Protease colored yellow and Polymerase RdRP colored lilac), as is characteristic of iflaviruses. The “rhv_like” protein domains of VP2 and VP3 are highlighted in black, as well as the “CRPV_capsid” protein domain of the VP1 protein is colored in light lilac at 10%. Below the representation of the polyprotein, the variation in GC content (guanine and cytosine) throughout the genome of our viral sequence is highlighted.

From a Mosquito virome library (B20), a complete genome with a length of 9709 nucleotides was obtained, which we named Mosq_B20_SP01. The genome obtained presents a characteristic organization of the *Iflaviridae* family and its representatives, as shown in Figure 1.

We used the ORFfinder tool (https://www.ncbi.nlm.nih.gov/orffinder/, accessed on 11 March 2024), we identified that the Mosq_B20_SP01 genome has a positive orientation and single strand, presenting a single large ORF (with a start codon at position 603 and a stop codon at position 9566), which encodes a polyprotein of 2987 amino acids. Using a BLASTX search, we obtained the “max score” result: 2865, referring to the polyprotein of *Calumiyane virus* (GenBank accession number QRW42874.1), e-value equal to 0.0, coverage of 91% and percentage of identity equal to 49%. The BLASTX “best hit” sequence corresponds to an isolate CSM002_20a (GeneBank accession number MW434114.1), classified as belonging to the Family Iflaviridae, obtained from a mosquito of the genus Culiseta captured in late autumn 2017 in the State of California in the States United States of America [3]. The amino acid sequence of the Mosq_B20_SP01 polyprotein was submitted to BLASTP, resulting again in the QRW42874.1 polyprotein as the “best hit”, with a “max score” of 2925, e-value equal to 0.0, coverage of 98% and identity percentage equal to 49.11%. The *Calumiyane virus* sequence, with a positive orientation and single strand, also presents a single ORF (with a start codon at position 749 and a stop codon at position 9703), encoding a polyprotein with a total of 2984 amino acids. Despite the similarity in amino acid sequences between Mosq_B20_SP01 and QRW42874.1, they are divergent with the distance calculated at 48.70%.

**Figure 2:**
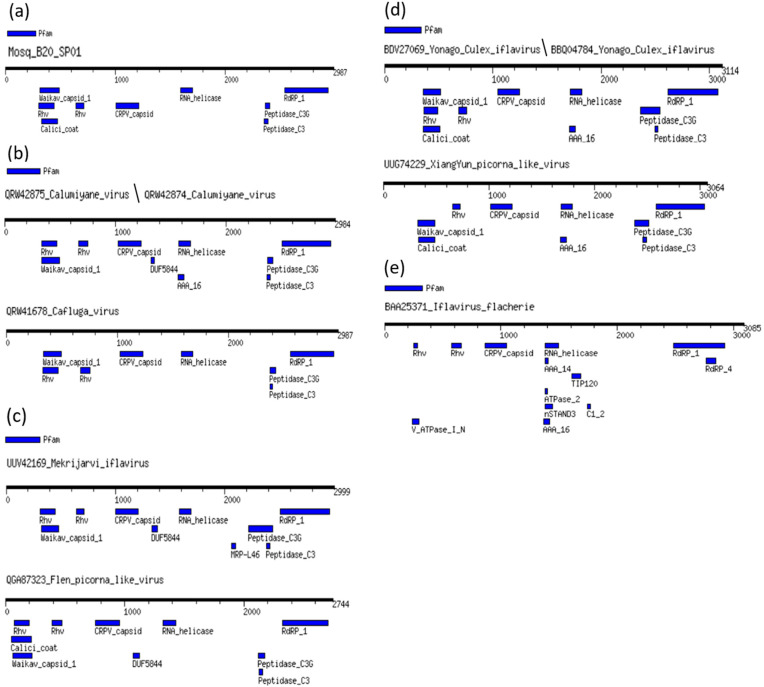
Protein domains of Mosq_B20_SP01 polyproteins compared to other iflaviruses. In (**a**–**d**) the main protein domains of viral sequences that infect culicidae are represented; in (**e**), the protein domains of the viral sequence of the type species that does not infect mosquitoes. The viral sequences that infect mosquitoes have many protein domains in common, and the sequence representing the type species presented a greater number of different protein domains compared to the viral sequences that infect mosquitoes.

We compared the domains of the different regions of the Mosq_B20_SP01 polyprotein, the BLASTP cognate sequence, *Calumiyane virus* (QRW42874), as well as the sequence of the type species: *Iflavirus flachiere* (BAA25371), from the iflavirus group and seven other sequences referring to associated viruses to iflaviruses that infect mosquitoes from the Culicidae family (Figure 2), to better elucidate the similarity between them. Domains were detected using the MotifFinder server and the Pfam database was used as a reference (https://www.genome.jp/tools/motif/, accessed 26 April 2024).

All translated sequences of polyproteins from viruses that infect mosquitoes, that is, leaving out the sequence of *Iflavirus falchiere* (type species), which infects silkworms, presented two protein domains of the Rhv type (except *XiangYun Picorna-like virus* that has a Rhv domain composed of amino acids from position 660 to 730), a Waikav_capisid_1 domain, a CRPV_capisid domain, an RNA_helicase domain, a Peptidase_C3G domain, a Peptidase_C3 domain, and an RdRP_1 domain. In (a), the sequence of the present work showing all protein domains in common with other viruses that infect mosquitoes, and the Calic_coat protein domain in common only with the two representatives of the *Yonago Culex iflavirus* species represented in (d); (b) indicates that the two representatives of *Calumiyane virus* as two domains DUF5844, and AAA_16, which are absent in the sequence of *Cafluga virus*, it is worth highlighting that this last domain, AAA_16, is also found in the two representatives of the species *Yonago Culex iflavirus* and *XiangYun Picorna-like virus*; in (c), the *Mekrijarvi iflavirus* sequence has an MRP_L46 domain that distinguishes it from *Fleen Picorna-like virus*, and both have a DUF5844 domain also present in the two representatives of *Calumiyane virus*; (d) demonstrates that the *XiangYun Picorna-like* virus sequence is distinguished from the two representatives of the *Yonago Culex iflavirus* species by lacking one of the two Rhv domains; finally, in (e), the sequence of *Iflavirus flacherie* presents the common domains found in all viruses that infect *culicidae*, except for the Waikav_capsid_1 domain absent in it; however, it presents the following protein domains absent in all sequences of culicidae-infecting viruses, V_ATPase_I_N, AAA_14, ATPase_2, nSTAND3 and C1_2, and finally, it has an AAA_16 domain in common only with the sequences of the two representatives of *Calumiyane virus*, as well as the two representatives of *Yonago Culex iflavirus*, and with the sequence of *XiangYun Picorna-like* virus.

However, Figure 2 demonstrates that the composition of protein domains predicted by the MotifFinder of Mosq_B20_sp01 is made up of protein domains characteristic of iflaviruses, with a composition more similar to that of viruses that infect mosquitoes.

**Figure 3:**
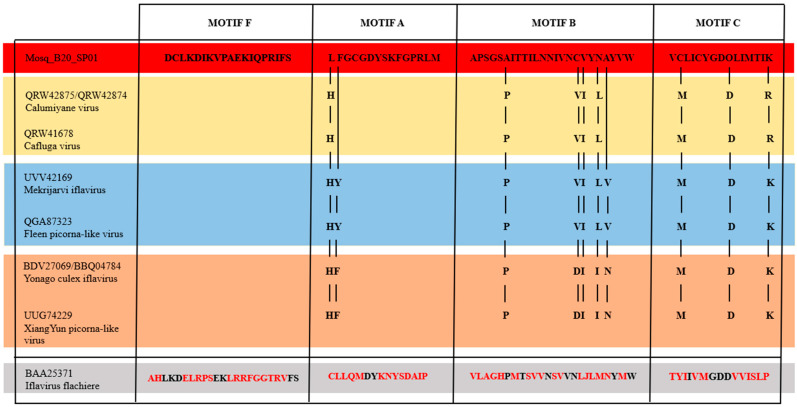
Comparison between motifs F, A, B and C of the RdRP protein domain. The vertical lines indicate the variations in the amino acid residue positions of the RdRP protein motifs between Mosq_B20_SP01 and the other mosquito-infecting sequences. Mosq_B20_SP01 and the other sequences that infect mosquitoes, demonstrated great similarity to each other, forming 4 groups highlighted in red colors (Mosq_B20_SP01, infects mosquitoes of the genus *Anopheles*); light orange (BDV27069, BBQ04784, UUG74229—genus *Culex*); light blue (UVV42169, QGA87323—genus *Ochlerotatus*); and light gold (QRW42875, QRW42874 and QRW41678—genus *Culiseta*). The *Iflavirus flachiere* sequence, highlighted in gray, showed great divergence in its amino acid residues (divergent residues highlighted in red) of the RdRP protein motifs in relation to the residues of the sequences that infect mosquitoes.

We also compared, according to Figure 3, the motifs within the genomic regions of RNA-dependent RNA Polymerases (RdRP), from Mosq_B20_SP01, with other sequences that infect mosquitoes (QRW42875/QRW42874—Blast cognates, and QRW41678, UVV42169, QGA87323, BDV27069, BBQ04784, UUG74229), and the *Iflavirus flachiere* type species sequence (BAA25371).

All protein motifs “F”, “A”, “B” and “C” of the polyprotein sequences among the viruses that infect mosquitoes of the *Culicidae* Family were conserved, with few changes; however, in comparison with the same protein motifs of the polyprotein sequence of the type species virus *Iflavirus flachiere*, showed great divergence. All viruses that infect mosquitoes have their “F” Motifs without changes in the amino acid sequences. Still among the viruses that infect culicidae, the “C” motif presented the same amino acid sequence among them, except for the two viruses that infect mosquitoes of the genus *Aedes* (*Mekrijarvi iflavirus* and *Fleen picorna-like virus*), and the virus represented by our query Mosq_B10_SP01, these showed the change in the last position of the “C” motif from an “R” amino acid residue to a “K” amino acid residue. Still, regarding mosquito-infecting viruses of the genus *Aedes*, they also presented two more exclusive amino acid residues in positions 2 of the “A” motif and 21 of the “B” motif, which are the “Y” and “V” residues, respectively. Therefore, the three viruses that infect mosquitoes of the genus *Culex*, two *Yonago culex iflavirus* and one *XiangYun picorna-like virus*, presented a set of three exclusive residues in positions 17, 20 and 21 (amino acid residues D, I and N, respectively). The sequence Mosq_B20_SP01, found in mosquitoes of the genus *Anopheles*, presents two characteristic residues in positions 1 of the “A” motif and 18 of the “B” motif, residues “L” and “V”, respectively.

### 3.3. Phylogenetic Trees

**Figure 4:**
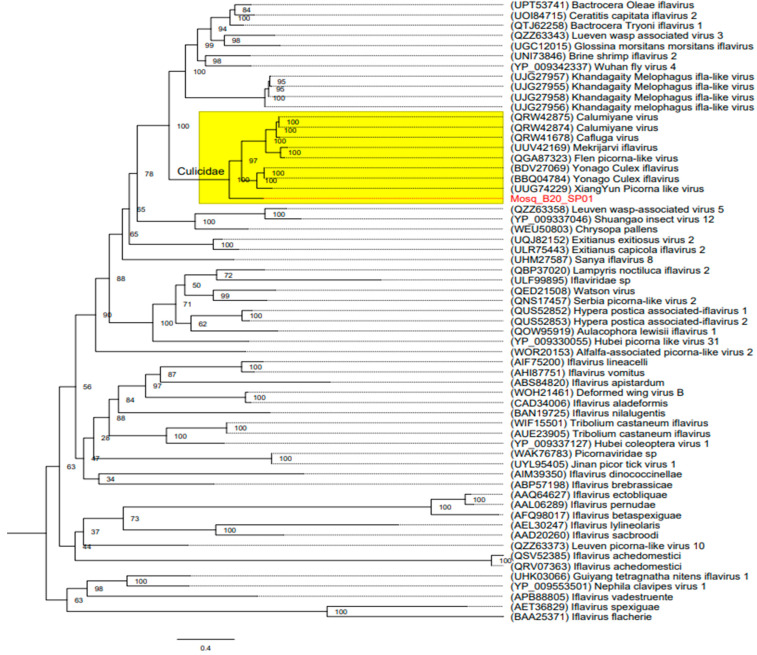
Maximum likelihood phylogenetic tree constructed based on amino acid sequences from the RdRP region. The bar corresponds to the nucleotide diversity along the branch of the tree. The virus obtained in this investigation is shown in Red (Mosq_B20_SP01). The putative new virus clustered, strongly based on the high bootstrap of 100, into a distinct clade representing a group of mosquito-infecting iflaviruses of the *Culicidae* family, represented in Yellow.

The phylogenetic tree inferred was produced using amino acid sequences from the RdRP region (Figure 4), from our query (Mosq_B20_SP01), from the cognate sequence of *Calumiyane virus* (GenBank accession number: PQRW42874), with a better score than BLASTp, a Type Species sequence of *Iflavirus flachiere* (GenBank accession number: BAA25371), plus 17 iflavirus species reference sequences obtained from the GenBank database (accession numbers: QSV52305, QRV07363, APB88805, AIF75200, AIM39350, AHI87751, AFQ98017, AET36829, AEL30247, ABS84820, ABP57198, AAQ64627, CAD34006, AAL06289, AAD20260, BAN19725, QRW42875), and 41 iflavirus-Associated Virus sequences (accession numbers: QRW4167 8, UUV42169, QGA87323, BDV27069, BBQ04784, UUG74229, QZZ63343, UPT53741, QTJ62258, UOI84715, UJG27857, UJG27956, UJG27958, UJG27955, UNI73846, YP_009342337, UGC12015, UQJ82152, QOW95919, 52, QUS52853, ULR75443, QBP37020, QED21508, QNS17457, QZZ63358, WEU50803, YP_009337046, UHM27587, YP_009330055, QZZ63373, ULF99895, UHK03066, WOR20153, YP_009553501, WAK76783, UYL95405, YP_009337127, WIF15501, AVE23905, WOH21461), with coverage of at least 50% in BLASTp. We also inferred trees using nucleotide sequences of the near-full-length genome and the capsid region of iflaviruses (Figure S1).

### 3.4. Genetic Diversity

**Figure 5:**
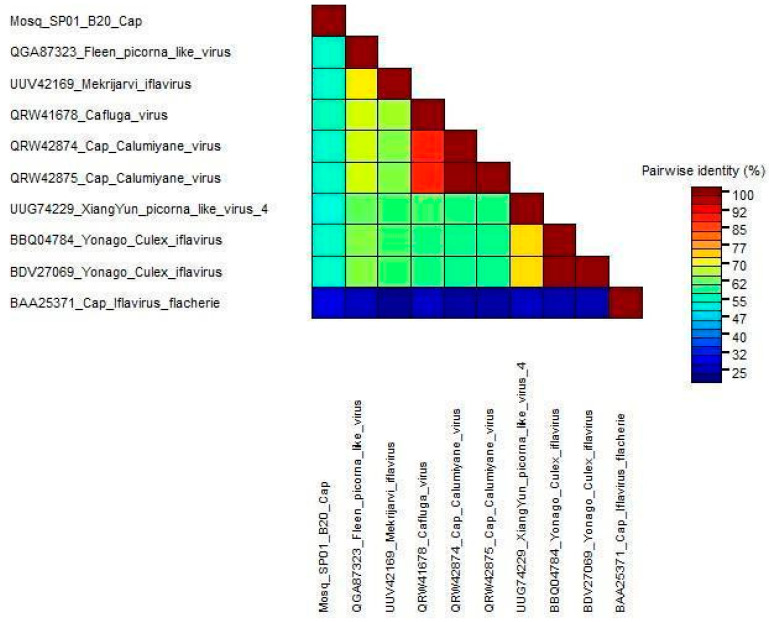
The identities of the translated sequences of the capsid regions were obtained using the SDT Program version 1.2. Estimation of similarity alignments for each unique pair of sequences was performed using algorithms implemented in MUSCLE.

We used the nucleotide identity of the complete genomes of Mosq_B20_SP01 and other viruses with culicidae hosts to compare genetic diversity. In general, the paired identities of iflaviruses infecting culicidae hosts and the putative new iflavirus obtained in this study ranged from 57.05% to 63.68%. We also performed the same for the translated sequences of the capsid regions for the same group of sequences mentioned above, and the identities in relation to our query varied from 26.54% to 53.84%, as shown in Figure 5, thus being below the minimum limit of 90% required as a criterion by the ICTV for it to be considered as belonging to the same species in relation to the compared sequences. Therefore, Mosq_B20_SP01 possibly corresponds to a new species of iflavirus. We also applied the identity matrix to the helicase and capsid region of these viruses (Figure S2).

### 3.5. Host Analysis

**Figure 6:**
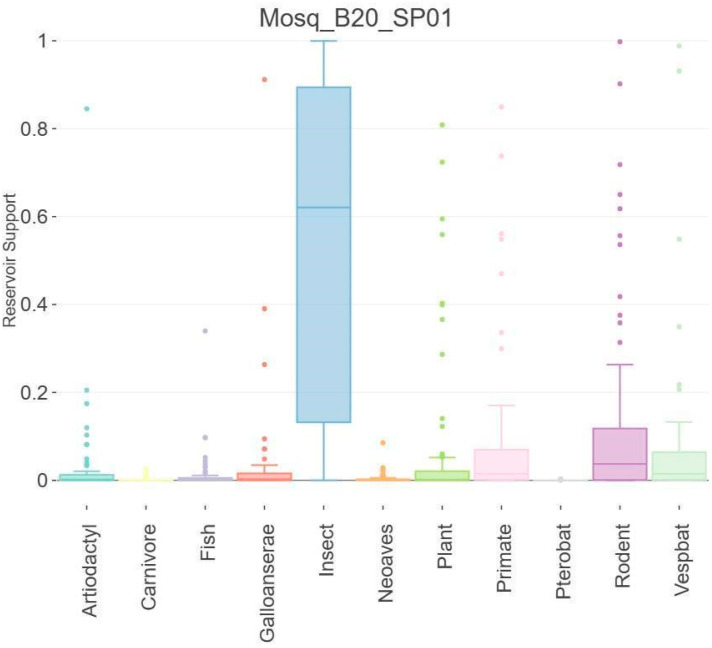
Host prediction of Mosq_B20_SP01; Viral Host Predictor was performed. The analysis indicates that the supposed new iflavirus (Mosq_B20_SP01) has tropism for arthropod hosts of the Insecta class.

For host prediction of Mosq_B20_SP01, the Viral Host Predictor was performed, which uses the gradient boosting machine algorithms developed by Babayan et al. (2018) [44] to predict reservoir hosts, arthropod vectors and virus transmission status RNA transmitted by arthropods. Algorithm predictions are based on host-associated biases in viral genomes (“genomic biases”) and phylogenetic relatedness to viruses with known transmission ecology (“phylogenetic neighborhood”). Figure 6 suggests that Mosq_B20_SP01 has an affinity for insect hosts.

In this study, we characterized the genome of a new species of iflavirus. The sequence, designated as Mosq_B20_SP01, exhibits a positive-sense single-strand RNA with one large open reading frame (ORF) encoding a polyprotein of 2987 amino acids. A BLASTX search identified the closest match as the *Calumiyane virus* polyprotein (GenBank QRW42874.1) with a max score of 2865, 91% coverage, and 49% identity. The best hit was an isolate, CSM002_20a (GenBank MW434114.1), classified under the Iflaviridae family from a mosquito in California. A subsequent BLASTP search confirmed the QRW42874.1 polyprotein as the best match, with a max score of 2925, 98% coverage, and 49.11% identity. Despite the high similarity, the sequences diverged with a distance of 48.70%. Mosq_B20_SP01’s protein domains are characteristic of iflaviruses, closely matching those of mosquito-infecting viruses. The RNA-dependent RNA Polymerase (RdRP) motifs “F,” “A,” “B,” and “C” were conserved with few changes. Motif “C” was identical except for variations in *Mekrijarvi iflavirus*, *Fleen picorna-like virus*, and Mosq_B20_SP01, which showed an amino acid change from “R” to “K.” Unique residues were identified in the “A” and “B” motifs of viruses infecting *Aedes* and *Culex* mosquitoes, with specific residues in Mosq_B20_SP01 found in *Anopheles* mosquitoes. A phylogenetic tree was constructed using RdRP region amino acid sequences from Mosq_B20_SP01, *Calumiyane virus* (GenBank PQRW42874), *Iflavirus flachiere* (GenBank BAA25371), 17 iflavirus species, and 41 iflavirus-associated viruses with at least 50% BLASTp coverage. This comprehensive comparison and phylogenetic analysis highlighted the genetic and evolutionary relationships of Mosq_B20_SP01 with other iflaviruses, demonstrating its closer affinity to mosquito-infecting iflaviruses.

## 4. Conclusions

The considerable similarity in genomic structure, conserved protein domains of the polyprotein, and protein motifs of the RNA-dependent RNA polymerases (RdRPs) between Mosq_B20_SP01 and known iflaviruses, along with a strong phylogenetic relationship with a group closely associated with mosquito hosts of the *Culicidae* family, led us to infer that Mosq_B20_SP01 belongs to the *Iflaviridae* family. Despite this, the relatively low identity presented by our query, when compared to the iflavirus sequences used in this study, does not meet the identity criterion above 90% required by the International Committee on Taxonomy of Viruses (ICTV) for classification within the same species. Consequently, Mosq_B20_SP01 can be considered a new species of iflavirus, which we have provisionally named *Iflavirus desanae*.

The discovery of *Iflavirus desanae* represents a significant expansion of our knowledge regarding the diversity of viruses that circulate among various mosquito species in Brazil. This finding underscores the complexity and richness of the viral ecosystem within mosquito populations and highlights the importance of ongoing surveillance and research in understanding the virome of vector species. Furthermore, it opens new avenues for studying the interactions between viruses and their mosquito hosts, which could have implications for mosquito-borne disease dynamics and control strategies.

Identifying *Iflavirus desanae* also contributes to the broader field of virology by adding to the catalog of known iflaviruses and providing a new reference point for comparative genomic and evolutionary studies. It underscores the value of using advanced genomic tools and bioinformatics analyses to uncover previously unknown viral species and to elucidate their relationships with existing ones. As we continue to explore the genetic diversity of viruses in mosquito populations, we may uncover more novel viruses, each contributing unique insights into the mechanisms of virus evolution, host adaptation, and transmission.

In summary, the characterization of Mosq_B20_SP01 as a new species of iflavirus, *Iflavirus desanae*, highlights the ongoing evolution and diversity of viruses in mosquito populations. This discovery not only enriches our understanding of the *Iflaviridae* family but also emphasizes the need for continued research in mosquito virology to better understand the dynamics of virus–host interactions and their potential impact on public health.

## Data Availability

The original contributions presented in the study are included in the article/supplementary material, further inquiries can be directed to the corresponding author.

## Supplementary Materials

The following supporting information can be downloaded at: https://www.mdpi.com/article/10.3390/microorganisms12091749/s1, Figure S1: Phylogenetic trees; Figure S2: Identity matrix of iflaviruses.
