# Supplementary material for: New Iflavirus Species Characterized from Mosquitoes Captured in the Sao Paulo Zoological Facilities
Source: Microorganisms. 2024 Aug 23;12(9):1749. doi: 10.3390/microorganisms12091749 (PMC11434248; doi:10.3390/microorganisms12091749)
Supplement: Supplementary file 1 [file microorganisms-12-01749-s001.zip › microorganisms-3118797-supplementary.pdf]

Phylogenetic trees

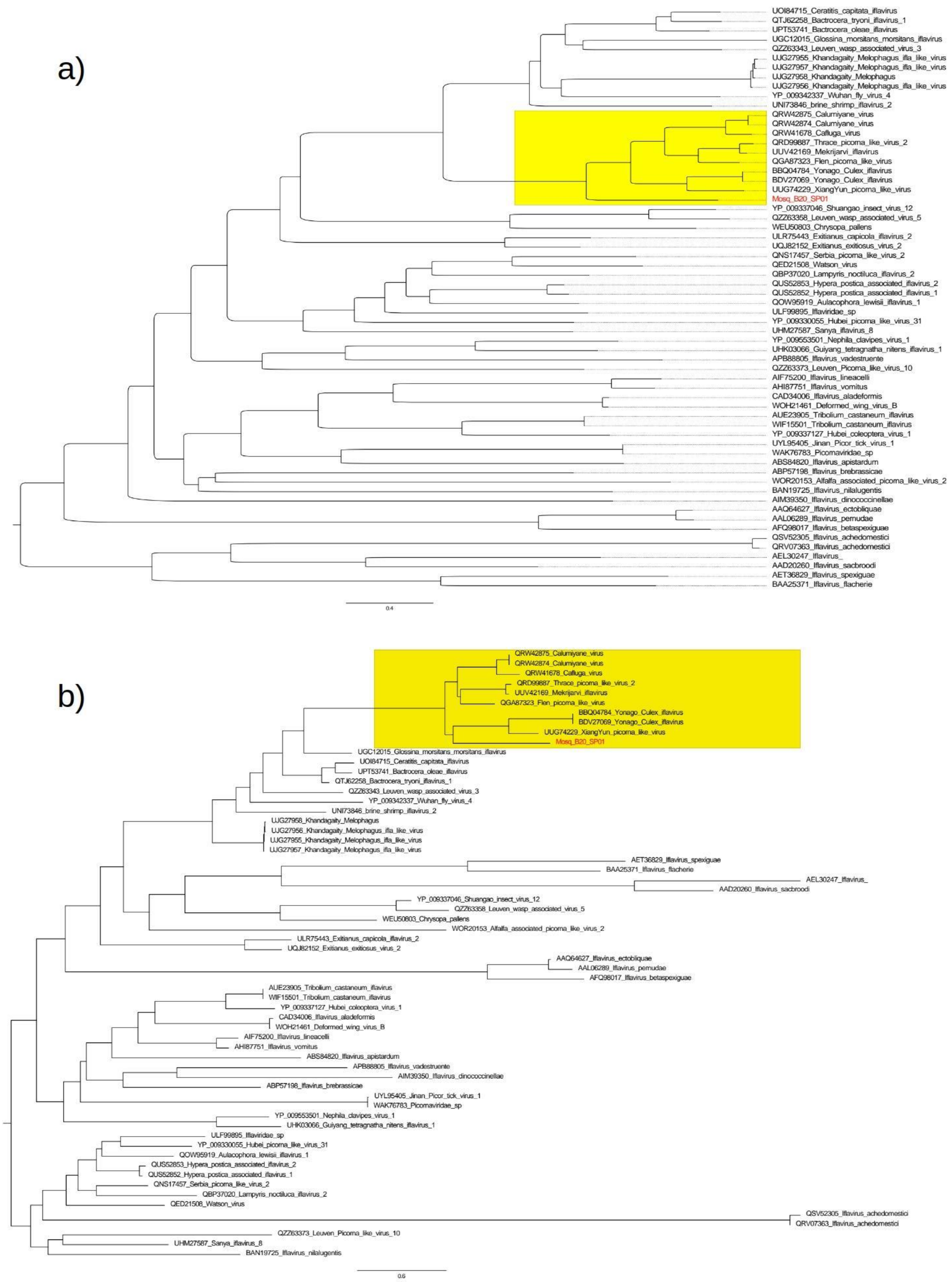

Figure S1. Maximum likelihood trees. Phylogenetic tree constructed using the near-full length genomes (a). Phylogenetic tree constructed using the Capsid region of iflaviruses (b). The bar corresponds to the nucleotide diversity along the branch of the tree. The virus obtained in this investigation is shown in Red (Mosq_B20_SP01). The putative new virus clustered into a distinct clade representing a group of mosquito-infecting iflaviruses of the *Culicidae* family, represented in Yellow.

Identity matrix

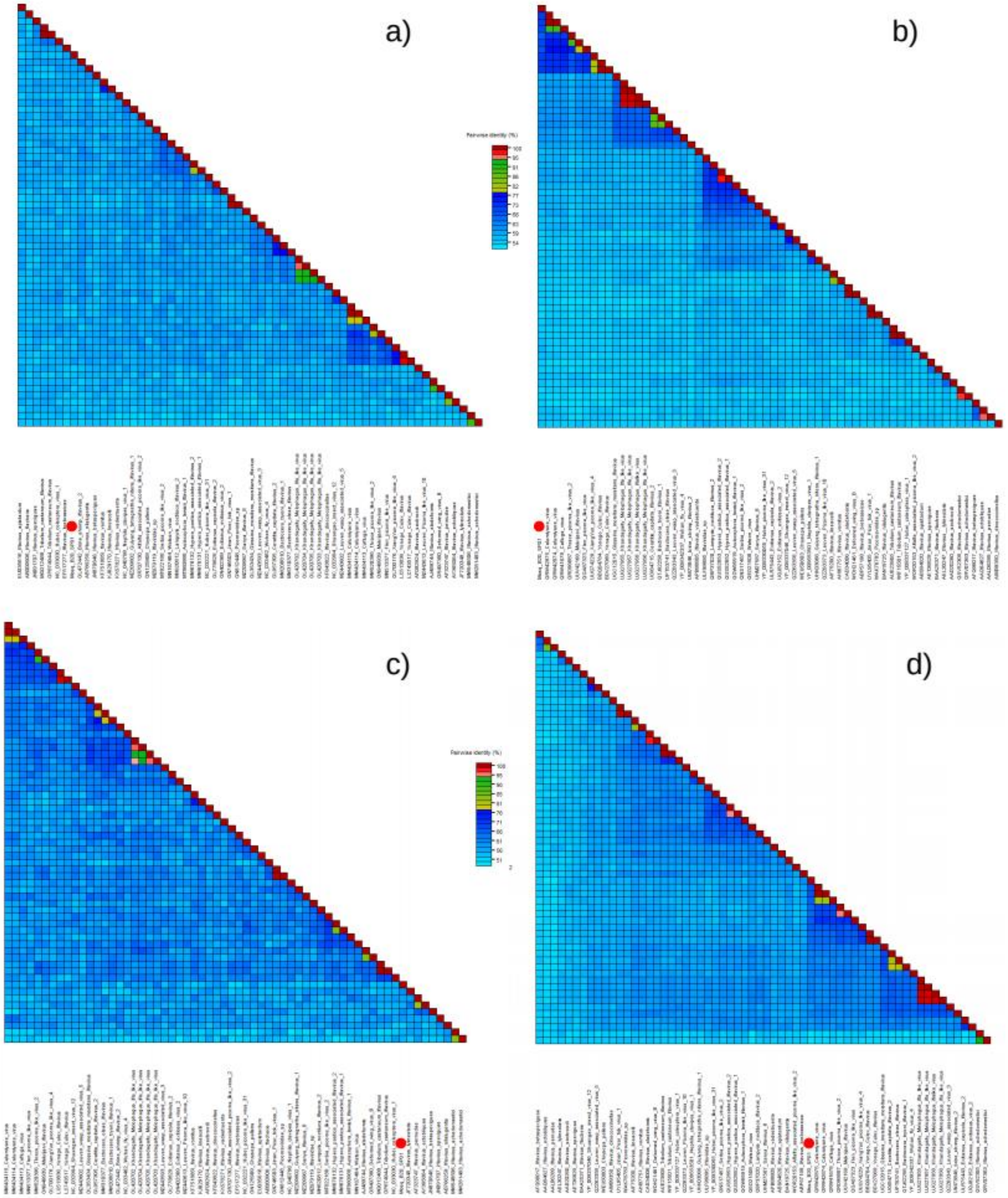

Figure S2. Nucleotide identity of helicase region of iflaviruses (a). Amino acid identity of helicase region of iflaviruses (b). Nucleotide identity of capsid region of iflaviruses (c). Amino acid identity of capsid region of iflaviruses (d). The virus obtained in this investigation is shown in Red (Mosq_B20_SP01).
